# What happened to anti-malarial markets after the Affordable Medicines Facility-malaria pilot? Trends in ACT availability, price and market share from five African countries under continuation of the private sector co-payment mechanism

**DOI:** 10.1186/s12936-017-1814-z

**Published:** 2017-04-25

**Authors:** ACTwatch Group, Louis Akulayi, Angela Alum, Andrew Andrada, Julie Archer, Ekundayo D. Arogundade, Erick Auko, Abdul R. Badru, Katie Bates, Paul Bouanchaud, Meghan Bruce, Katia Bruxvoort, Peter Buyungo, Angela Camilleri, Emily D. Carter, Steven Chapman, Nikki Charman, Desmond Chavasse, Robyn Cyr, Kevin Duff, Gylsain Guedegbe, Keith Esch, Illah Evance, Anna Fulton, Hellen Gataaka, Tarryn Haslam, Emily Harris, Christine Hong, Catharine Hurley, Whitney Isenhower, Enid Kaabunga, Baraka D. Kaaya, Esther Kabui, Beth Kangwana, Lason Kapata, Henry Kaula, Gloria Kigo, Irene Kyomuhangi, Aliza Lailari, Sandra LeFevre, Megan Littrell, Greta Martin, Daniel Michael, Erik Monroe, Godefroid Mpanya, Felton Mpasela, Felix Mulama, Anne Musuva, Julius Ngigi, Edward Ngoma, Marjorie Norman, Bernard Nyauchi, Kathryn A. O’Connell, Carolyne Ochieng, Edna Ogada, Linda Ongwenyi, Ricki Orford, Saysana Phanalasy, Stephen Poyer, Justin Rahariniaina, Jacky Raharinjatovo, Lanto Razafindralambo, Solofo Razakamiadana, Christina Riley, John Rodgers, Andria Rusk, Tanya Shewchuk, Simon Sensalire, Julianna Smith, Phok Sochea, Tsione Solomon, Raymond Sudoi, Martine Esther Esther, Katherine Thanel, Rachel Thompson, Mitsuru Toda, Chinazo Ujuju, Marie-Alix Valensi, Vamsi Vasireddy, Cynthia B. Whitman, Cyprien Zinsou, Sarah Tougher, Kara Hanson, Catherine Goodman

**Affiliations:** 1 Population Services International, 1120 19th St NW Suit 600, Washington, DC 20036 USA; 2 London School of Hygiene and Tropical Medicine, 15-17 Tavistock Place, London, WCH 9SH UK

**Keywords:** Artemisinin combination therapy, Private sector, Case management, Global Fund, Malaria treatment

## Abstract

**Background:**

The private sector supplies anti-malarial treatment for large proportions of patients in sub-Saharan Africa. Following the large-scale piloting of the Affordable Medicines Facility-malaria (AMFm) from 2010 to 2011, a private sector co-payment mechanism (CPM) provided continuation of private sector subsidies for quality-assured artemisinin combination therapies (QAACT). This article analyses for the first time the extent to which improvements in private sector QAACT supply and distribution observed during the AMFm were maintained or intensified during continuation of the CPM through 2015 in Kenya, Madagascar, Nigeria, Tanzania and Uganda using repeat cross-sectional outlet survey data.

**Results:**

QAACT market share in all five countries increased during the AMFm period (p < 0.001). According to the data from the last ACTwatch survey round, in all study countries except Madagascar, AMFm levels of private sector QAACT availability were maintained or improved. In 2014/15, private sector QAACT availability was greater than 70% in Nigeria (84.3%), Kenya (70.5%), Tanzania (83.0%) and Uganda (77.1%), but only 11.2% in Madagascar. QAACT market share was maintained or improved post-AMFm in Nigeria, Tanzania and Uganda, but statistically significant declines were observed in Kenya and Madagascar. In 2014/5, QAACT market share was highest in Kenya and Uganda (48.2 and 47.5%, respectively) followed by Tanzania (39.2%), Nigeria (35.0%), and Madagascar (7.0%). Four of the five countries experienced significant decreases in median QAACT price during the AMFm period. Private sector QAACT prices were maintained or further reduced in Tanzania, Nigeria and Uganda, but prices increased significantly in Kenya and Madagascar. SP prices were consistently lower than those of QAACT in the AMFm period, with the exception of Kenya and Tanzania in 2011, where they were equal. In 2014/5 QAACT remained two to three times more expensive than the most popular non-artemisinin therapy in all countries except Tanzania.

**Conclusions:**

Results suggest that a private sector co-payment mechanism for QAACT implemented at national scale for 5 years was associated with positive and sustained improvements in QAACT availability, price and market share in Nigeria, Tanzania and Uganda, with more mixed results in Kenya, and few improvements in Madagascar. The subsidy mechanism as implemented over time across countries was not sufficient on its own to achieve optimal QAACT uptake. Supporting interventions to address continued availability and distribution of non-artemisinin therapies, and to create demand for QAACT among providers and consumers need to be effectively implemented to realize the full potential of this subsidy mechanism. Furthermore, there is need for comprehensive market assessments to identify contemporary market barriers to high coverage with both confirmatory testing and appropriate treatment.

**Electronic supplementary material:**

The online version of this article (doi:10.1186/s12936-017-1814-z) contains supplementary material, which is available to authorized users.

## Background

The private sector is key to the provision of malaria treatment in many countries in sub-Saharan Africa, constituting a substantial proportion of the overall market [1], and tending to reach the poorest segments of many societies [2]. However, in many cases, sub-Saharan African private sector anti-malarial markets have been characterized by a predominance of anti-malarial medicines that are banned or no longer recommended, including non-artemisinin therapies and artemisinin monotherapies [1]. Uptake of the World Health Organization’s recommended first-line treatment for uncomplicated malaria, artemisinin-based combination therapy (ACT) [3], has in the past been limited by a lack of consumer access [1, 4], and when available, high relative costs outside of the public sector [5, 6]. Inadequate access to prompt and effective treatment continues to contribute to malaria deaths in endemic countries of sub-Saharan Africa; of the 438,000 global deaths from malaria in 2015, 90% reportedly occurred in the region [7].

In order to increase utilization of the most effective treatments for malaria, the Affordable Medicines Facility-malaria (AMFm) was established by the Global Fund to Fight AIDS, Tuberculosis and Malaria (the Global Fund, hereafter) in 2010 with the aim of increasing uptake of quality-assured ACT medicines (QAACT) and decreasing use of artemisinin monotherapies. The AMFm aimed to (1) increase affordability, (2) increase availability and (3) increase use of QAACT, and (4) to crowd out artemisinin monotherapies. The AMFm consisted of nine pilots in eight countries (Cambodia, Ghana, Kenya, Madagascar, Niger, Nigeria, Uganda, and Tanzania mainland and Zanzibar) (although Cambodia was not included in the independent evaluation due to implementation delays). The AMFm aimed to achieve its four goals through negotiating QAACT price reductions from manufacturers, and subsidizing their prices through co-payment, administered at the manufacturer level. Supporting interventions for the subsidy programme included behaviour change communications (BCC), the training of private sector vendors, and the introduction of recommended retail prices for QAACT. All Global Fund subsidised QAACT packaging carried a green leaf logo which was promoted in demand creation activities as an indication of quality and affordable anti-malarial treatment.

An independent evaluation of the AMFm in 2012 demonstrated substantial increases in availability and market share, and large price decreases for QAACT in six out of eight pilots. The results of the AMFm evaluation have been described in detail elsewhere [8–10]. Briefly, the benchmark of a 20% point increase in QAACT availability was met in five out of the eight pilots. The benchmark of a 10% point increase in QAACT market share was met in four pilots, with a further three having weak statistical evidence. Finally, the benchmark of QAACT prices falling below three times the price of the most popular non-ACT anti-malarial in the country was met in five pilots. When applied to the private sector alone, the independent evaluation’s conclusions regarding the success metrics still hold. Positive market shifts were found to be largely due to changes in the private for-profit sectors in pilot countries [9]; indeed, the subsidy facility was described as a ‘game changer’ in the private for-profit sectors of all but two countries by the independent evaluators [8]. A systematic review of the literature examining the effects of anti-malarial subsidies likewise found subsidies to be successful in increasing availability and reducing costs of ACT. Furthermore, improved availability and affordability tended to be equitable between rural and urban areas, and across income gradients [11]. The AMFm evaluation also found this to be the case in several pilots [8]. Following the AMFm period, the Global Fund continued a QAACT subsidy programme termed the private sector co-payment mechanism (CPM). The CPM has been in operation at national scale in six countries since the end of the AMFm pilot and the subsequent transition period in 2013. However, there is no published evidence to-date on the effectiveness of the CPM. This paper addresses that gap by examining post-pilot evidence in five countries: Nigeria, Kenya, Madagascar, Tanzania and Uganda.

### Description of the private sector co-payment mechanism

Following on the AMFm pilot phase from 2010 to 2011, the programme of subsidies and price negotiations continued in six countries: Ghana, Kenya, Madagascar, Nigeria, Tanzania and Uganda. Initial support for the subsidy provided by AMFm Phase 1 donors (i.e., Bill and Melinda Gates Foundation, UK Department for International Development [DFID], Government of Canada and UNITAID) continued during a transition period, until implementation of a mechanism funded by the Global Fund called the CPM for ACT. The grant-funded CPM may now be included as part of a country’s malaria funding application to the Global Fund.

**Fig. 1:**
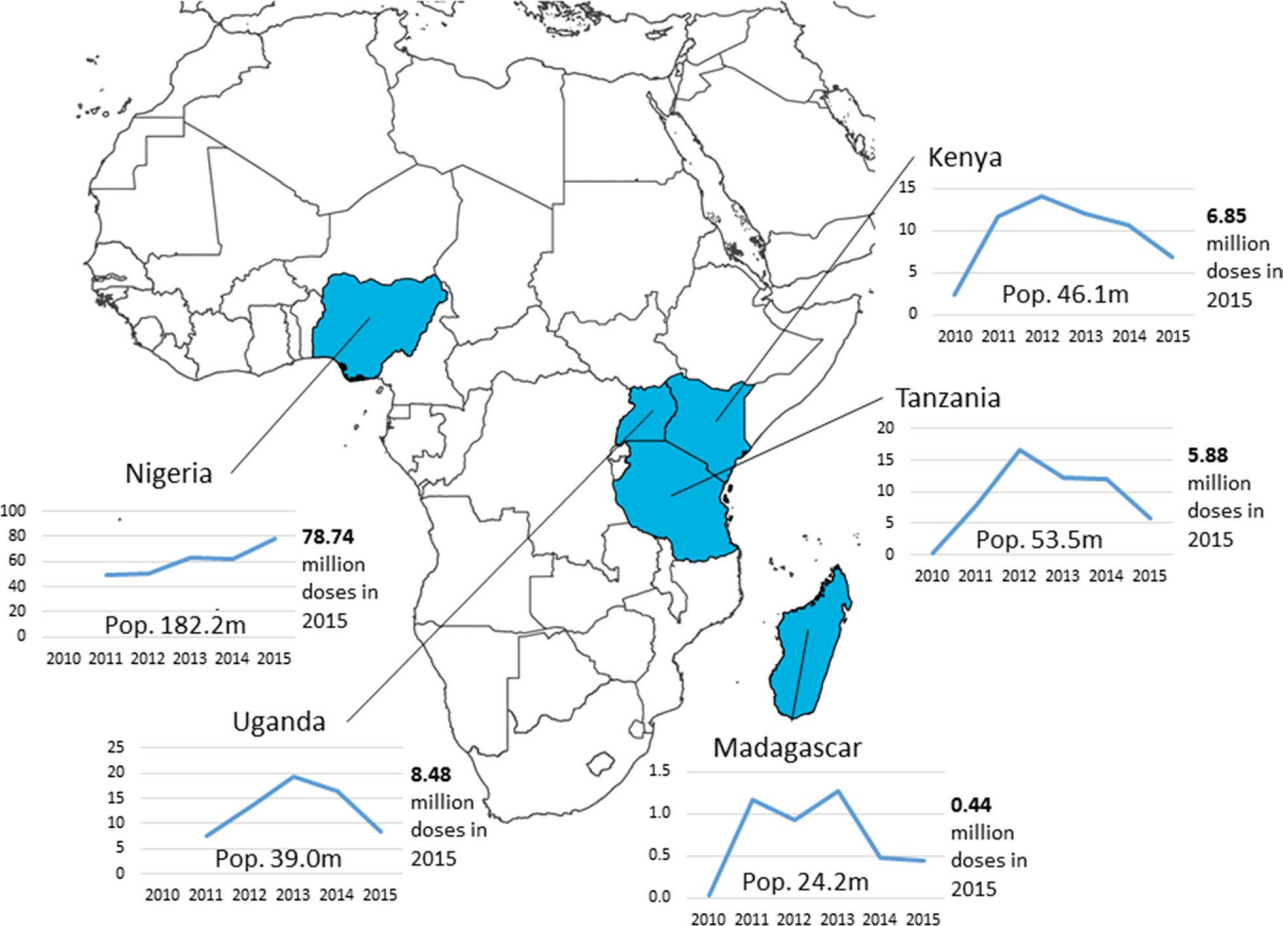
Quantity of co-paid ACT delivered to private sector first-line buyers, 2010–2015

Figure 1 summarizes quantities of co-paid ACT delivered to the private sector during the AMFm period and the CPM through 2015 within the five countries included in this study (all CPM countries except Ghana), and references the population size of each country [12] (Personal Communication, Global Fund Sourcing Department 2016). Within each country, the peak for delivery of co-paid doses occurred after the evaluated pilot period, 2015 in Nigeria, or in 2012 or 2013 for the other four countries.

The CPM operated using the three key elements of the AMFm: price negotiations with manufacturers, ACT subsidies at ‘factory gate’, and additional supporting interventions. The CPM focuses exclusively on the private for-profit sector supply of QAACT given that the independent evaluation showed that AMFm had greater impact on the supply of QAACT in the private than the public sector. Prior to the AMFm period, the Global Fund was a primary source of funding for QAACT within the public sector of pilot countries [9], and this funding support for the public sector continued post-AMFm outside of the CPM. The CPM therefore complemented Global Fund support to the public sector by providing a mechanism for improving access to QAACT in the private sector with the aim of reaching the large proportion of the population in participating countries seeking fever treatment in this sector.

**Fig. 2:**
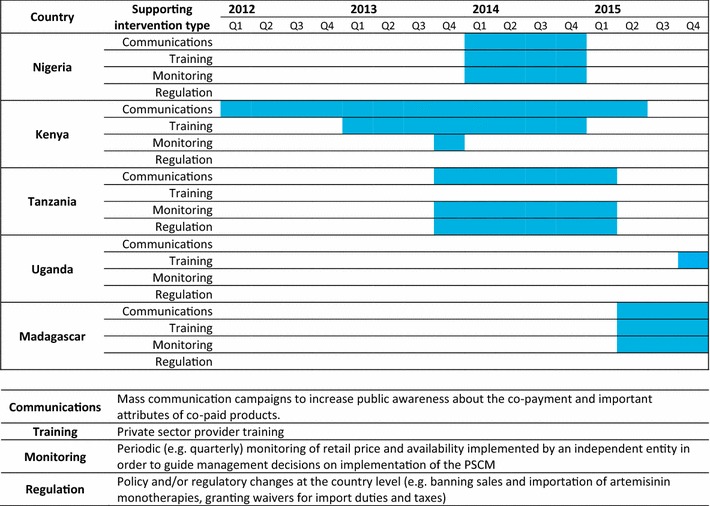
Timeline of CPM supporting interventions implementation

The extent to which supporting interventions were implemented within each country post-AMFm varied greatly. Mass communication campaigns, private provider training, independent monitoring of retail price and availability, and policy and/or regulatory changes were implemented at various time points in the five countries examined here (Fig. 2) (Personal Communication, National Malaria Control Programmes).

The AMFm was characterized by a high degree of centralized management by a dedicated Global Fund Secretariat. In the transition to the CPM, many aspects that had been centrally managed and controlled under the AMFm were devolved to national level under the responsibility of the Global Fund Principal Recipient (PR) with guidance from a national co-payment task force. Key changes in the mechanism that occurred in the transition from AMFm to CPM are detailed in Table 1.

**Table 1:** Key features of the AMFm and CPM. Source: Personal Communication, The Global Fund, 24/08/2016

	AMFm	CPM
Oversight responsibilities	A sub-committee of the Global Fund Board provided oversight of the Secretariat	The Country Coordinating Mechanism at country level provided oversight of implementation by the PR of the grant
Subsidy level	Set by the Global Fund Secretariat and applied across all pilots. The subsidy level under the AMFm was targeted at 95%, but in practice it differed by formulation and pack size	Since 2013, countries have set their own subsidy levels and these have evolved over time
Supplier management	Supplier management was handled centrally under the AMFm and the CPM	
Price negotiations	A maximum price approach was outsourced to a negotiating agent	A competitive tender was managed by the Sourcing Department at the Global Fund Importantly, this resulted in a supplier- and product-specific reduction in prices of about 30%. This reduction has offset the falls in subsidy levels in some countries
First-line buyer responsibilities	The Global Fund determined first line buyer eligibility to participate and sent Local Fund Agents to conduct spot checks to monitor compliance with the terms and conditions of the agreement	PRs assessed first-line buyers, determined eligibility, maintained and oversaw agreements, and conducted spot checks for compliance with the terms and conditions of the agreement
Order approvals	The Global Fund AMFm Unit approved all orders, initially on demand, and then on a monthly basis	One to four rounds for ordering per calendar year per country, and the PR had to review, validate and approve each round of co-payment allocations before the Global Fund could notify manufacturers of orders approved for co-payment
Monitoring and evaluation	The Global Fund commissioned an independent evaluation and price-tracking surveys	PRs were responsible for tracking price and availability in the private sector

With devolution of certain components of the CPM to country level, there was more room for variation between countries in subsidy levels, and greater flexibility to respond to local anti-malarial market conditions. Table 2 outlines changes in subsidy levels that occurred within each country post-pilot through 2015. Madagascar was the only AMFm country in this study to maintain the 95% subsidy for first-line buyers. Elsewhere, the subsidy level was reduced to as low as 70% in Kenya and Uganda.

**Table 2:** Subsidy levels for the CPM. Source: Personal Communication, The Global Fund, 24/08/2016

Nigeria	Early to mid-2013: changed to 85% subsidy for all pack sizes
Kenya	Early to mid-2013: changed to 70% subsidy for all pack sizes
Tanzania	Early 2014: changed to 80% adult and 90% paediatric subsidy levels Early 2015: changed to 75% adult and 85% paediatric subsidy levels.
Uganda	Early 2014: changed to 50% adult and 70% paediatric subsidy levels Mid- 2015: changed to 70% subsidy for all pack sizes
Madagascar	Maintained the 95% subsidy for all pack sizes

The extent to which any of the successes identified in the independent evaluation of the AMFm have been maintained or improved upon with implementation of the CPM is unreported in the literature to-date. The aim of this paper is to analyse what happened to QAACT market share, availability and price in the private for-profit sector during the period following the AMFm. Specifically, we use ACTwatch outlet survey data to examine to what extent the changes observed during the 2010–2011 evaluated period of the pilot were maintained with implementation of the CPM through 2014/15 in five countries, and where they were maintained, whether there have been significant improvements in those indicators.

## Methods

ACTwatch was launched in 2008 by Population Services International (PSI) in collaboration with the London School of Hygiene and Tropical Medicine with support from the Bill and Melinda Gates Foundation. The goal of the project was to generate timely, relevant and high quality evidence about anti-malarial markets for policy makers, donors and implementing organisations. As of 2016, ACTwatch had gathered data from a total of 12 malaria endemic countries in sub-Saharan Africa and the Greater Mekong Sub-region. This paper presents data from outlet surveys in five sub-Saharan countries that took part in the AMFm pilot. It excludes data from other countries that were not part of the AMFm pilot. Detailed ACTwatch project and methodological information have been published elsewhere [13, 14].

### Design and sampling

ACTwatch outlet surveys are nationally-representative cross-sectional quantitative surveys conducted among a sample of outlets stocking anti-malarial medicines and diagnostics. Surveys were repeated over time to inform, monitor and evaluate policies and strategies designed to improve access and use of malaria diagnostics and first-line treatments. A detailed description of the ACTwatch outlet survey methods is available elsewhere [13]. Briefly, all categories of outlets with the potential to stock anti-malarials in both the public and private sector were included in the study. In the public sector, this included government and non-government not-for-profit health facilities (hospitals, centres, clinics and posts) and community health workers. Outlets sampled in the private sector included private for-profit health facilities (hospitals, centres and clinics), pharmacies, drug stores (registered/regulated and unregistered/unregulated), general retailers selling fast-moving consumer goods, and itinerant drug vendors (mobile vendors without a fixed service delivery point).

Lists of all potentially eligible outlets were not routinely available and therefore a cluster sampling approach with an outlet census was used to identify outlets for inclusion. Clusters were administrative units ideally with a typical size of 10,000–15,000 inhabitants, and were selected using probability proportional to population size sampling. Within each selected cluster all outlet types with the potential to provide anti-malarials to consumers were screened, with anti-malarial audits completed in all outlets found to have one or more anti-malarials in stock the day of the survey.

Boundaries for the outlet census were typically extended to larger administrative units for the census of public health facilities and pharmacies, in order to over-sample these relatively uncommon but important outlet types.

Each study was stratified to deliver estimates for relevant research domains. All countries had urban and rural stratification, with the exception of Nigeria in 2009, 2013 and 2015, for which six geopolitical zones were used as research domains. Each study round was powered to detect a minimum of a 20% point change in QAACT availability among anti-malarial stocking outlets between each round and within each domain at the 5% significance level with 80% power. The number of study clusters was calculated for each research domain based on the required number of anti-malarial stocking outlets and assumptions about the number of anti-malarial stocking outlets per cluster. Sample size requirements for follow-up surveys were calculated using information from previous survey rounds including anti-malarial and QAACT availability, outlet density per cluster, and design effect.

Data collection periods varied by country and over time but were typically during the peak malaria transmission season for each country and lasted between 6 weeks and 2 months. Efforts were made to ensure surveys were implemented over similar time points across the survey rounds.

### Training and fieldwork

Interviewer training consisted of standardized classroom presentations and exercises as well as a field exercise. Exams administered during training were used to select data collectors, supervisors, and quality-controllers, who received additional training. Data collection teams were provided with a list of selected clusters and official maps that illustrated administrative boundaries. In each selected cluster, fieldworkers conducted a full enumeration of all outlets that had the potential to provide anti-malarials. This included enumeration of outlets with a physical location, as well as identification of community health workers and itinerant drug vendors using local informants. The primary provider/owner of each outlet was invited to participate in the study and screening questions were administered to assess anti-malarial availability. Interviews were conducted in the local language using questionnaires that were translated from English to the local language and back to English to confirm translations. All surveys were paper-based with the exception of Madagascar 2015 and Uganda 2015, where data were collected using Android phones and forms created using DroidDB (^©^SYWARE, Inc., Cambridge, MA, USA). Quality control measures implemented during data collection included questionnaire review by supervisors and interview verification visits conducted by quality controllers to between 10 and 20% of all outlets. Any discrepancies that were found were resolved. Double data entry was conducted using Microsoft Access (Microsoft Corporation, Redmond, WA, USA) with built-in range and consistency checks.

### Information collected on anti-malarials

The outlet survey questionnaire included an audit of all available anti-malarial medicines. Providers were asked to show the interviewer all anti-malarial medicines currently available. A product audit sheet captured information for each unique anti-malarial product in the outlet, including formulation, brand name, active ingredients and strengths, package size, manufacturer and country of manufacture. Providers were asked to report the retail price for each medicine as well as the amount distributed to individual consumers (as opposed to wholesale purchasers) in the last week.

### Data analysis

Data were analysed across survey rounds using Stata (StataCorp College Station, TX) and R (^©^The R Foundation, Vienna, Austria). Standard indicators were constructed according to definitions applied across the ACTwatch project and have been described in detail elsewhere [1, 14]. Briefly, anti-malarials identified during the outlet drug audit were classified according to information on drug formulation, active ingredients and strengths as non-artemisinin therapies, artemisinin monotherapies and ACT. Non-artemisinin therapies were classified as sulfadoxine pyrimethamine (SP) or other non-artemisinin therapies. Although no longer indicated for malaria case management, SP is still purchased for case management and recommended in all study countries for intermittent preventive therapy of malaria during pregnancy (IPTp). Artemisinin monotherapies were further classified as oral and non-oral, the latter including medicines recommended for the first-line treatment of severe malaria. ACT were classified as QAACT or non-QAACT. QAACT were ACT granted World Health Organization (WHO) prequalification, ACT in compliance with the Global Fund Quality Assurance Policy, on the Global Fund list of approved pharmaceutical products for procurement, or ACT granted regulatory approval by the European Medicines Agency (EMA). Classification was completed by matching product audit information (formulation, active ingredients, strengths, manufacturer, country of manufacture and package size) to the most recent lists of approved medicines available from the WHO, Global Fund, and EMA for each survey round.

Anti-malarial market composition was defined as the percentage breakdown of anti-malarial outlets by type, with anti-malarial outlets defined as all those with anti-malarials in stock on the day of the survey. QAACT availability is presented out of those outlets that had anti-malarials in stock. Significant differences in QAACT availability levels between years in each country were estimated using logistic regression, with a binary dependent variable for availability of QAACT at the outlet level, and a dummy independent variable for year.

To calculate market share, anti-malarial sales were standardized to allow meaningful comparisons between anti-malarials with different treatment courses and different formulations. The adult equivalent treatment dose (AETD) was defined as the amount of active ingredient required to treat an adult weighing 60 kg according to WHO treatment guidelines [3]. Provider reports on the amount of the drug sold or distributed during the week preceding the survey were used to calculate volumes in AETDs according to type of anti-malarial. Measures of volume included all dosage forms to provide a complete assessment of anti-malarial market share. The statistical significance of differences in QAACT market share was estimated using Stata’s ratio command, and the post-estimation ‘lincom’ (linear combination) command.

Median private sector price per AETD was calculated for QAACT and for the most popular non-artemisinin therapy in the most recent round, SP. The interquartile range (IQR) is presented as a measure of dispersion. Price data were collected in local currencies and deflated to 2009 US dollar prices using national consumer price indices and published exchange rates for the period of data collection. While all QAACT are by definition tablet formulations, SP may be available in other formulations including syrups and injections. Price measures included tablet anti-malarials only, given differences in unit costs for tablet and non-tablet formulations. Statistical significance for year on year differences in median price in each country was estimated in R, using the Mann–Whitney–Wilcoxon test. Because this test provides a measure of relative rank, rather than strictly testing for the difference between two medians, it is possible that significant differences will be identified when there is no difference between the medians themselves [15]. This therefore would represent a difference in the price distribution between 2 years, rather than directly a difference between two medians.

Sampling weights were calculated as the inverse of the probability of cluster selection. All point estimates were weighted using survey settings and all standard errors calculated taking account of the clustered and stratified sampling strategy with the relevant suite of survey commands in each statistical package.

## Results

A total of 139,738 outlets were screened to assess availability of anti-malarial medicines across the five countries and 18 survey rounds between 2009 and 2015. An audit of all available anti-malarials was completed in 34,441 outlets. In total, 242,541 anti-malarial drugs were audited. Table 3 shows a detailed breakdown of the screening and audit results for each survey round.

**Table 3:** Results of the outlet census and anti-malarial audit by country and survey year

Country	Year	Screened (N)	Outlets the met screening criteria	Anti-malarial audit complete (N)	Anti-malarials audited (N)	QA ACT audited (N)
West & Central Africa						
Nigeria	2009	5456	2210	2113	20,841	1192
	2011	7938	1567	1486	13,391	2119
	2013	5148	1828	1714	14,358	4799
	2015	13,483	3624	3473	33,532	9581
East Africa						
Kenya	2010	13,897	2625	1888	8376	2052
	2011	11,383	2112	1854	9544	3669
	2014	12,676	2477	2133	9899	3234
Tanzania	2010	3120	710	624	5544	416
	2011	3702	799	787	9701	2045
	2014	4724	2160	2129	17,307	4905
Uganda	2010	11,153	2590	2410	14,427	2893
	2011	16,207	3285	3138	20,283	5495
	2013	7932	3504	3307	19,777	7182
	2015	9438	4780	4328	26,640	7380
Southern Africa						
Madagascar	2010	6769	2642	2414	5579	1790
	2011	10,046	2854	2360	7234	3233
	2013	10,149	2021	1756	6101	3851
	2015	13,481	1361	1040	3170	1501

**Fig. 3:**
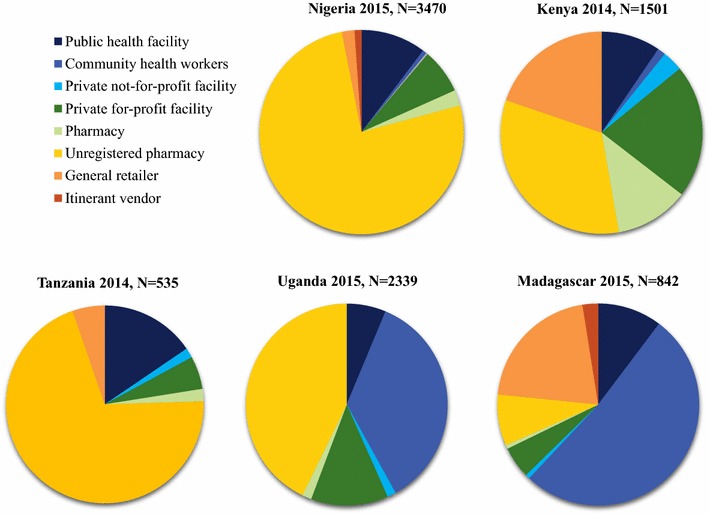
Anti-malarial market composition by country for the most recent survey round

The private sector constituted the majority of service delivery points for malaria treatment provision in four out of the five countries. Figure 3 shows that in terms of absolute number of places where anti-malarial medicines were available, most were categorized as private for-profit outlets. Only Madagascar had a majority of outlets in the public/not-for-profit sector, where community health workers represented 51.7% of all outlets, and a large majority, therefore, of all public sector outlets. The private sector nevertheless represented a substantial proportion of the anti-malarial market in Madagascar. In the other four countries, drug stores tended to be the most numerous outlet type among all those providing malaria treatment. In Nigeria and Tanzania, drug stores accounted for more than half of all anti-malarial service delivery points (76.0 and 70.2%, respectively). Private for-profit health facilities and pharmacies tended to be less numerous, although they accounted for a substantial proportion of all anti-malarial stocking outlets in Kenya (21.4 and 11.8%, respectively). The extent to which general retailers were part of the anti-malarial market varied, from Uganda where general retailers were not involved in anti-malarial distribution, to Kenya and Madagascar where about one in five anti-malarial stocking outlets were general retail shops (19.8% in Kenya; 20.9% in Madagascar). In Kenya, this means that nearly one in four (23.0%) private sector outlets providing anti-malarials were general retailers, and in Madagascar, over half (56.0%) of anti-malarial stocking private sector outlets were general retail outlets. Itinerant drug vendors with anti-malarials in stock were uncommon across all countries, although found in both Madagascar and Nigeria (2.5 and 1.1% of all outlets stocking anti-malarials, respectively).

### Private sector QAACT availability

**Fig. 4:**
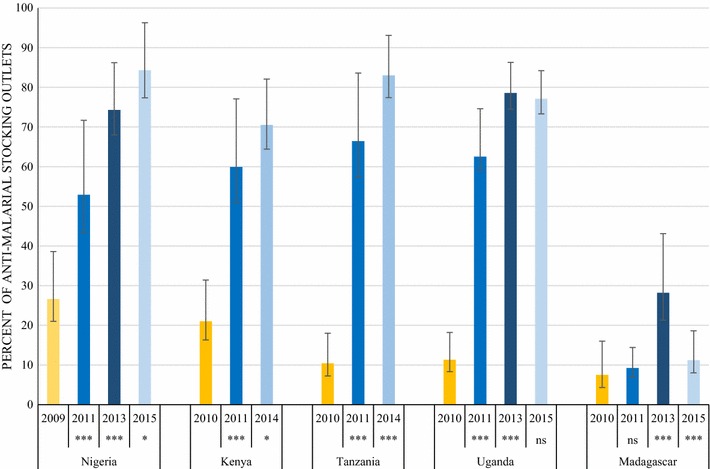
Availability of QAACT among private sector anti-malarial stocking outlets. Significant difference in QAACT availability between the round indicated and the previous round: *ns* not significant, * p < 0.05, *** p < 0.001

Figure 4 summarizes the availability of QAACT among anti-malarial stocking private sector outlets pre- (2009/2010) and post-(2011) AMFm, and subsequently during implementation of the CPM. QAACT availability in the most recent survey round was greater than 70% in Nigeria, Kenya, Tanzania and Uganda. The highest level was found in Nigeria, where QAACT availability exceeded 80% in 2015 (84.3%). Madagascar had a substantially lower level of QAACT availability, at 11.2% in 2015.

Significant increases were found in availability between pairs of consecutive survey rounds in all countries for at least one post-2011 time period. In Nigeria, QAACT availability rose by 21% points in the period 2011–2013 (p < 0.001) and then by a further 10% points through 2015 (p < 0.05). Kenya and Tanzania also experienced statistically significant increases in availability of 10% points (p < 0.05) and 16% points (p < 0.001) respectively between 2011 and 2014. In Uganda, a significant increase in availability was observed between 2011 and 2013, and this level of availability (over 75%) was also maintained in the most recent survey round (no significant difference). In Madagascar, there was no significant rise in availability during AMFm, however QAACT availability increased by 19% points between 2011 and 2013 (p < 0.001), but decreased significantly to near pre-AMFm levels in 2015 (p < 0.001).

### Private sector anti-malarial market share

**Fig. 5:**
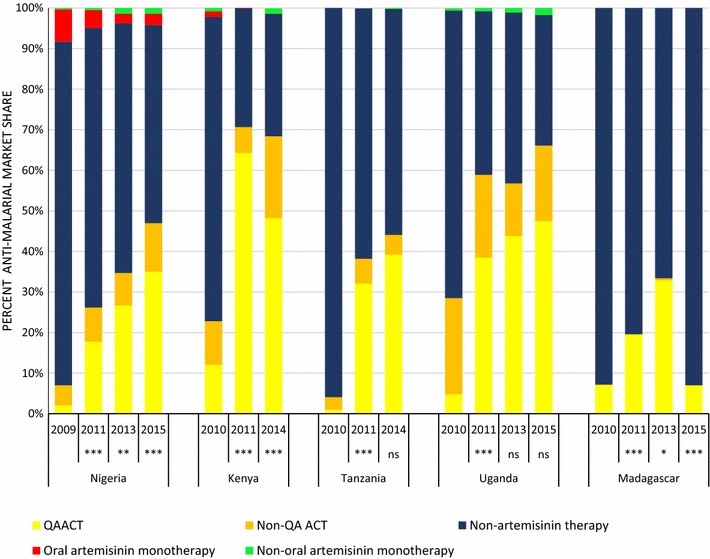
Private sector market share by anti-malarial type. Significant difference in QAACT market share between the round indicated and the previous round: *ns* not significant, * p < 0.05, ** p < 0.01, *** p < 0.001

Figure 5 summarizes QAACT anti-malarial market share in the private sector pre- (2009/2010) and post- (2011) AMFm, and subsequently during implementation of the CPM. Market share is also shown for non-QAACT, non-artemisinin therapies and artemisinin monotherapies. The share of private for-profit anti-malarial distribution accounted for by QAACT varied across countries, but was less than 50% in all countries. In the most recent survey round, it was highest in Kenya and Uganda (at 48.2 and 47.5%, respectively), followed by Tanzania (39.2%) and Nigeria (35.0%). QAACT market share was lowest in Madagascar (7.0%) in 2015.

QAACT market share in all five countries increased during the AMFm period (p < 0.001). In both Tanzania and Uganda, QAACT market share increases were maintained (no significant change compared with post-2011 levels), while in Nigeria there were further statistically significant increases in QAACT market share between survey rounds (p < 0.001 between 2011-2013 and 2013-2015). Kenya saw a statistically significant decrease in QAACT market share between 2011 and 2014 (p < 0.001). However, ACT market share in Kenya remained similar between 2011 and 2014 with non-QAACT appearing to have displaced QAACT during this period. In Madagascar, initial post-AMFm gains in QAACT market share (p < 0.05) had been eliminated by the most recent survey round, with large, significant declines (p < 0.001), returning to pre-AMFm levels.

Data within each type of private sector outlet show that QAACT market share improvements in the case of Nigeria, Tanzania and Uganda, and declines in Kenya and Madagascar were not necessarily uniform across all outlet types (Additional file 1).

Non-artemisinin therapies accounted for about one-third of all anti-malarials distributed in the private sector during the most recent survey rounds in Kenya (30.2%) and Uganda (32.0%), almost half in Nigeria (48.8%) and over half in Tanzania (55.7%) and Madagascar (93.6%). The most common type of non-artemisinin therapy distributed in each country’s private sector was SP.

Oral artemisinin monotherapy was found consistently only in Nigeria’s private sector. Market share for oral artemisinin monotherapy in Nigeria fell significantly between 2011 and 2013 (p < 0.001), and did not increase significantly between 2013 and 2015. Nevertheless, oral artemisinin monotherapy accounted for 2.5% of market share in the most recent survey round.

### Private sector price

**Fig. 6:**
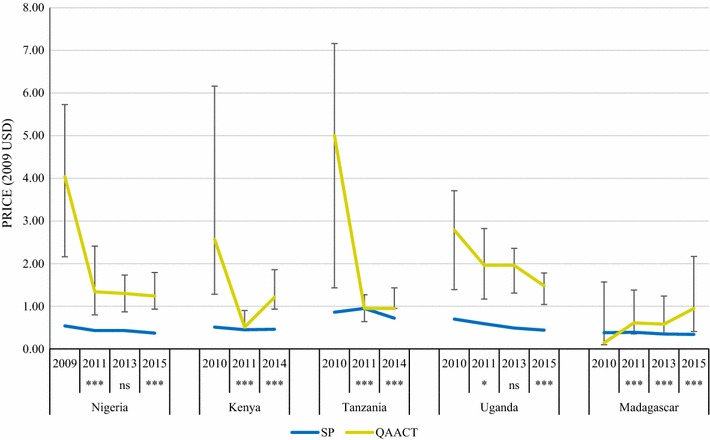
Median private sector price for one AETD of QAACT and SP in 2009 USD. Significant difference in QAACT price between the round indicated and the previous round: *ns* not significant, * p < 0.05, *** p < 0.001

Four of the five countries experienced significant decreases in median QAACT price during the AMFm period (to 2011). Post-AMFm pilot, these prices were maintained in Nigeria and Uganda to 2013 and then underwent a decline in both countries between 2013 and 2015 (p < 0.001). In Tanzania, the median price of QAACT remained the same between 2011 and 2014, however the distribution of prices, as illustrated in the upwards shift in IQR, resulted in a statistically significant test results for price trend (p < 0.001). Price increased significantly in Kenya between 2011 and 2014 (p < 0.001), although remained consistently below pre-AMFm levels. The price of QAACT in Madagascar rose during the AMFm period, and despite small but significant price declines between 2011 and 2013 (p < 0.001), QAACT price increased between 2013 and 2015 (p < 0.001) (Fig. 6). This rise was likely due to large price increases per AETD of artemether-lumefantrine (AL) in Madagascar in that period, as artesunate-amodiaquine (ASAQ) price remained stable.

SP prices were consistently lower than those of QAACT in both the AMFm and CPM periods, with the exception of Kenya and Tanzania in 2011, where they were equal. Price differences between SP and QAACT have narrowed considerably since 2009/2010. In the most recent survey round, QAACT and SP prices were most similar in Tanzania (in 2014), when QAACT remained 1.3 times more expensive than SP. In all other countries, QAACT were two to three times more expensive than SP during the most recent survey round (Nigeria, 3.4; Kenya, 2.6; Uganda, 3.4; Madagascar, 2.8) (Additional file 2).

**Fig. 7:**
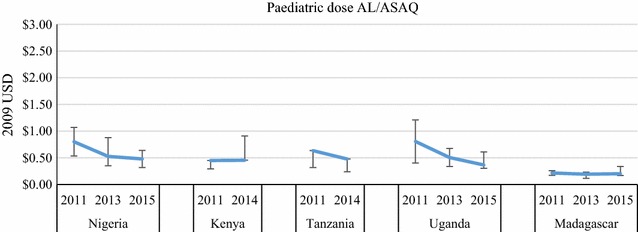
Package price for paediatric dose AL or ASAQ, in 2009 USD

**Fig. 8:**
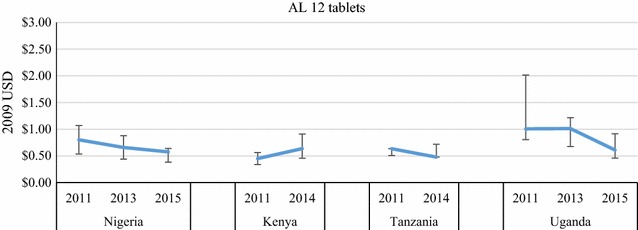
Package price for 12 tablets of AL, in 2009 USD

**Fig. 9:**
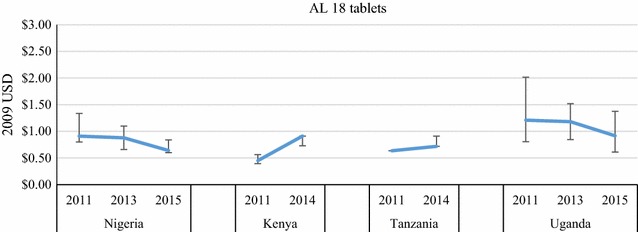
Package price for 18 tablets of AL, in 2009 USD

**Fig. 10:**
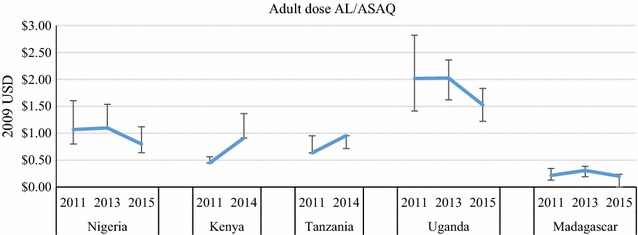
Package price for one adult dose of AL or ASAQ, in 2009 USD

Price data were disaggregated by package size for the most common QAACT type in each country (ASAQ in Madagascar, AL in the other four countries) (Figs. 7, 8, 9, 10). Post-AMFm falls in prices seen at aggregate level in Uganda and Nigeria clearly occurred across all package sizes. Post-AMFm increases in price in Kenya and Tanzania were observed for larger pack sizes, but not for AL 6-tablet pack in both countries, and the AL 12-tablet pack in Tanzania.

## Discussion

This paper examined whether the successes of the AMFm in improving availability, market share and price of QAACT between 2010 and 2011 [9] were maintained or improved upon with continuation of a private sector co-payment mechanism administered by the Global Fund through 2014/15. The results are particularly significant given the importance of the private sector in distributing anti-malarial drugs in these countries [1, 4]. While encouraging, findings also highlight a need for further improvements in QAACT uptake in the private sector.

The improvements in anti-malarial markets observed in this study occurred in the context of an evolution in the copayment mechanism, from highly centralized management under the controlled conditions of the time-limited AMFm pilot to decentralization of management and oversight to the national level in the CPM period. Each country set new subsidy levels, most often reducing the subsidy for first-line buyers, with the exception of Madagascar where the high subsidy level under AMFm was maintained. Supporting interventions were largely not systematically implemented across countries during the CPM period, with the exception of reported mass communications regarding the subsidy in Kenya. Implementation strength of supporting interventions was not measured. The number of co-paid ACT delivered to the private sector peaked after the period covered by the independent evaluation of AMFm in each country, indicating that demand and successful resupply for first-line buyers continued under the decentralized CPM model. These findings demonstrate operational effectiveness under sustained implementation outside of a tightly controlled pilot.

### Post-AMFm anti-malarial market improvements

In the post-AMFm period, there were statistically significant increases in private sector QAACT availability in Kenya, Madagascar, Nigeria, Tanzania and Uganda, but with a subsequent fall in the case of Madagascar. Furthermore, the vast majority of private sector outlets with anti-malarials in stock had QAACT in stock during each survey round in all countries except Madagascar. The majority of people seeking malaria treatment in these countries do so in the private sector, and these results indicate that throughout the CPM period, they were likely to find QAACT in these private outlets, whereas before AMFm implementation this was not the case. Furthermore, post-AMFm QAACT market share in the private sector was maintained or further increased in Nigeria, Tanzania and Uganda.

### Persistent challenges to increased QAACT uptake

Despite these broadly positive results for availability and market share, there was evidence of a persistent gap in the uptake of QAACT after 4–5 years of a private sector copayment mechanism (2010–2014/15). While it should be recognised that implementation varied over time and across countries, and that funding and subsidy levels fell after the initial pilot period, it was notable that QAACT market share in the private sector remained at less than 50% in each country. Non-artemisinin therapies, particularly SP, accounted for one-third to one-half of all anti-malarials distributed in Kenya, Nigeria, Tanzania and Uganda, and over 90% of anti-malarials distributed in Madagascar. Non-artemisinin therapies should continue to account for a small portion of anti-malarial market share in all of these countries because SP is recommended for IPTp [3]. However, persistent substantial SP market share is cause for concern. Continued uses of SP likely include management of fever/malaria in people of all ages given the widespread availability of products with packaging and patient instructions for uncomplicated malaria.

The continuing sub-optimal QAACT market share, despite high availability, highlights the need for expansion of supporting interventions to promote QAACT uptake. Persistent distribution of non-artemisinin therapies despite availability of ACT has been documented in a number of countries within public and private sectors and well before the AMFm pilot [16, 17]. Provider behaviour is one contributing factor to low ACT demand and use, and may reflect low levels of provider awareness about recommended first-line treatments and/or beliefs and preferences for non-first-line medicines [18–20]. Within the context of the AMFm, there is some evidence to suggest that communications campaigns implemented as part of the AMFm pilot may have been effective in increasing private provider knowledge regarding the first-line treatment [21]. However, awareness of official guidance is unlikely sufficient to drive ACT uptake alone and strategies are needed to influence not only provider knowledge but also provider preferences [22]. There is some evidence to suggest that trainings for providers in the informal private sector may have benefits [23]. Trainings targeting private providers were reportedly implemented as part of the CPM in Kenya and Nigeria, although evidence about implementation strength and effectiveness of these trainings is not available. There is need for additional evidence about private provider preferences and practices to inform appropriate strategies to facilitate changes in stocking and dispensing behaviours in the context of mechanisms such as the CPM.

Low QAACT uptake is also influenced by consumer demand [20]. There is a gap in the contemporary evidence base for information on factors influencing consumer treatment-seeking behaviour. While a number of studies concerning patient treatment-seeking behaviour were conducted and summarized in key reviews in the 1990s and early 2000s [24, 25], there is need for updated evidence, particularly in the context of large-scale investments and strategies to improve malaria case management in public and private sectors. The primary supporting intervention targeting consumers under the AMFm and CPM was mass media communications campaigns with a focus on promoting ACT with the green leaf logo and in some instances, the recommended retail price. These were implemented to varying degrees across countries, however evidence on implementation strength and effectiveness vis-à-vis QAACT uptake is not available. Mass media communications campaigns have been shown to play an important role in a range of health interventions, particularly when part of a wider set of activities [26]. Although behaviour change communication has been a critical component of malaria strategies, most programs have not been rigorously evaluated. Evidence is limited on effectiveness of various approaches and materials as well as on the overall effectiveness of communications for behaviour change in this context [27].

The relatively high cost of QAACT, despite reductions in price with AMFm and CPM interventions, may also be limiting QAACT update. SP remains less expensive than QAACT in all study countries, and QAACT were two to three times more expensive than SP during the most recent survey round in Kenya, Madagascar, Nigeria and Uganda. Reducing ACT retail price increases ACT use [6, 11], but results of this study suggest that retail prices achieved through the CPM model may not be low enough to achieve optimal uptake.

In addition to supporting interventions targeting provider and consumer behaviour, the policy and regulatory environment may influence the success of copayment mechanisms [24]. Strengthened policies and regulations may be needed to curtail the availability and distribution of artemisinin monotherapies for malaria case management [28].

Finally, the requirements for such supporting interventions are evolving in line with the goals of malaria case management in endemic country settings. WHO and national programme guidelines now place emphasis on confirmation of all suspected cases [29], meaning that there is need to re-evaluate the primary threats and specific barriers to case management in the private sector, and to develop locally tailored and global market-based approaches that will be cost-effective in ensuring not only use of appropriate and effective malaria treatments but also appropriate diagnosis.

### Market deteriorations

In contrast to the generally positive trends in key indicators in Nigeria, Tanzania and Uganda, results were mixed in Kenya and highly unfavourable in Madagascar. While QAACT availability in Kenya increased post-AMFm to just over 70%, market share significantly declined to less than 50% coinciding with a drop in subsidy level from 95% to 70%. At the same time, market share for non-QAACT increased such that one in five anti-malarials distributed in Kenya in 2014 were non-QAACT, indicating that QAACT were to some extent displaced by non-QAACT between 2011 and 2014 [30]. This is concerning given potential threats to patient health and malaria parasite clearance associated with using sub-standard medicines [31–35]. What appears to be a rising demand for and use of non-QAACT is particularly surprising given that Kenya reported implementing mass communications during the AMFm and continuing throughout CPM implementation. However, the reach and consistency of these communications has not been formally assessed and effectiveness has not been established. It is also interesting to consider if a more gradual reduction in subsidy level over time might have preserved some of the QAACT market share in Kenya despite the retail price increase that would be expected following a reduction in subsidy level. Evidence is needed to inform additional strategies to generate and sustain demand for quality-assured products.

In Madagascar, the AMFm yielded no significant improvements in private sector QAACT availability and significant but minimal improvements in private sector QAACT market share. Post-AMFm, QAACT market share and price increased to moderate levels between 2011 and 2013, but then declined to pre-AMFm levels by 2015. The CPM in Madagascar was halted between 2014 and late 2015 following an investigation by the Global Fund’s Office of the Inspector General, resulting in very few co-paid ACT being delivered before the end of 2015, with only one round of co-payments held at the end of that year. The country’s 2009–2014 political crisis also had limiting effects on the extent of external donors’ support for health programmes in the public sector, with health commodity stock-outs and overall weak national health infrastructure and commodity management systems continuing to pose challenges [36]. Furthermore, an interruption to major Global Fund-funded activities during this period resulted in widespread ACT stock-outs nationally. Further to these supply chain and health system problems, the supporting interventions relating to the CPM had not started as of the final survey round reported here (in 2015) (Personal Communication, The Global Fund). The relatively poor results in Madagascar can therefore perhaps be better understood as a reflection of poor implementation, rather than the failure of a well-implemented programme.

## Limitations

This study has several limitations. First, the complex nature of the CPM, operating at national level in several countries with heterogeneous anti-malarial distribution chains and markets has meant that there were no good controls at sub-national or national level to use as comparators in this analysis. The degree to which we could make any kind of causal claims as to the effect of the CPM is therefore limited. Nevertheless, seen in the context of the results of the AMFm independent evaluation, the evidence presented here from the post-pilot period is suggestive of continued effects of ‘factory gate’ subsidies on the QAACT markets in the countries where the mechanism was active. Furthermore, a systematic review of the literature examining the effects of subsidies on key ACT indicators suggested that in smaller-scale RCTs, there was strong causal evidence that these subsidies could be successful in reducing ACT prices and increasing their availability and market share in a range of settings [37]; this reinforces the findings presented here.

A further limitation is our reliance on self-report survey data. In each country’s outlet surveys, while anti-malarial audits were carried out by researchers, still depended on prices and sales volumes reported by the outlet supervisor. These responses were open to positive response bias. Differential levels of response bias for SP versus QAACT would not, however, necessarily be expected. It is possible though that there may be differential levels of response bias between survey rounds, as the emphasis placed on recommended retail price (RRPs) may have varied over time. As our focus has been on price difference between these two anti-malarials, rather than their absolute levels, the effect of this potential bias should be limited here. Using price per AETD permits price comparisons over time, and between types of anti-malarial drugs, but we acknowledge that this measure is less useful in understanding what consumers typically pay for treatment, as this varies according to various factors such as pack size.

## Conclusions

Improving the case management of malaria in the private sector is critical to improving coverage of appropriate malaria case management in many endemic countries. The results presented in this paper demonstrate that a private sector co-payment mechanism for QAACT, implemented at national scale for 5 years was associated with positive and sustained improvements in QAACT availability, price and market share in three out of five countries. These included the large West African country of Nigeria, with one of the highest malaria burdens in the world, as well as the East African countries of Uganda and Tanzania. Furthermore, substantial improvements in QAACT availability, price and market share observed during the AMFm period continued in these three countries as they transitioned to the CPM, characterized by decentralized management and national oversight of implementation. Mixed results were observed in Kenya, where post-AMFm QAACT market share was not maintained and QAACT appeared to have been displaced in part by non-QAACT following a reduction in the subsidy level while the quantity of co-paid QAACT delivered remained nearly constant. It is worth noting though that the QAACT market share remained well above pre-AMFm levels. In Madagascar, a number of contextual factors appear to have contributed to a lack of sustained market improvements under both the AMFm and CPM, including halted implementation of the programme. Results from this study suggest that a co-payment mechanism for QAACT targeting the private sector can be an effective and feasible strategy to improve availability and use of appropriate malaria treatment in malaria-endemic countries that have a substantial private sector market. However, results also demonstrate that this subsidy mechanism was not sufficient alone to drive QAACT uptake to 100%. Supporting interventions to address availability and distribution of non-artemisinin therapies and to create demand for QAACT among providers and consumers may be needed to realize the full potential of this type of subsidy mechanism. Furthermore, there is need for comprehensive market assessments in malaria endemic countries to identify contemporary market barriers to high coverage with not only appropriate malaria treatment but also confirmatory testing of all suspected cases.

## Additional files

**Additional file 1.** QAACT market share, as a proportion of the total anti-malarial sales within each outlet type, by year.

**Additional file 2.** Median private sector price for one AETD of QAACT and SP in 2009 USD, by country and year.

## Abbreviations

ACT: artemisinin-based combination therapy
AETD: adult equivalent treatment dose
AL: artemether-lumefantrine
AMFm: Affordable Medicines Facility for malaria
ASAQ: artesunate-amodiaquine
BCC: behaviour change communications
CPM: private sector copayment mechanism
DFID: Department for International Development
EMA: European Medicines Agency
IPTp: intermittent preventive therapy of malaria during pregnancy
IQR: interquartile range
N: number
PR: principal recipient
PSI: Population Services International
QAACT: quality-assured artemisinin combination therapy
RRP: recommended retail price
SP: sulfadoxine-pyrimethamine
USD: United States Dollar
WHO: World Health Organization
