# Supplementary material for: What happened to anti-malarial markets after the Affordable Medicines Facility-malaria pilot? Trends in ACT availability, price and market share from five African countries under continuation of the private sector co-payment mechanism
Source: Malar J. 2017 Apr 25;16:173. doi: 10.1186/s12936-017-1814-z (PMC5405529; doi:10.1186/s12936-017-1814-z)
Supplement: Supplementary file 1 — Additional file 1. QAACT market share, as a proportion of the total anti-malarial sales within each outlet type, by year. [file 12936_2017_1814_MOESM1_ESM.docx]

**Additional file 1: QAACT market share, as a proportion of the total anti-malarial sales within each outlet type, by year**

|  | **Private for-profit outlet** | | | | | | | | | | | |
| --- | --- | --- | --- | --- | --- | --- | --- | --- | --- | --- | --- | --- |
|  | **Health facility** | | **Pharmacy** | | **Drug store** | | **General retailer** | | **Itinerant vendor** | | **Total - all for-profit** | |
|  | Unweighted AETD within outlet type | % QAACT | Unweighted AETD within outlet type | % QAACT | Unweighted AETD within outlet type | % QAACT | Unweighted AETD within outlet type | % QAACT | Unweighted AETD within outlet type | % QAACT | Unweighted AETD within outlet type | % QAACT |
| Nigeria |  |  |  |  |  |  |  |  |  |  |  |  |
| 2009 | 12172.9 | 2.5% | 47224.5 | 3.7% | 39993.0 | 2.2% | 1711.4 | 0.8% | 65.1 | 0.0% | 101166.9 | 2.1% |
| 2011 | 2047.0 | 35.6% | 6906.5 | 11.1% | 53929.1 | 17.9% | 1564.2 | 9.1% | 532.7 | 1.1% | 64979.4 | 17.8% |
| 2013 | 2763.1 | 41.5% | 9800.8 | 16.6% | 42169.7 | 26.4% | 2030.5 | 30.0% | 58.7 | 4.6% | 56822.9 | 26.7% |
| 2015 | 4972.2 | 33.2% | 22957.5 | 22.9% | 98878.2 | 36.7% | 1711.5 | 19.8% | 193.7 | 15.1% | 128713.1 | 35.% |
| Kenya |  |  |  |  |  |  |  |  |  |  |  |  |
| 2010 | 4187.7 | 20.6% | 6993.6 | 26.7% | 10604.8 | 8.4% | 1682.6 | 0.0% | 3.0 | - | 23471.6 | 12.1% |
| 2011 | 5361.1 | 56.1% | 3182.2 | 45.1% | 18836.8 | 69.0% | 1336.7 | 49.1% |  | - | 28716.8 | 62.2% |
| 2014 | 4077.4 | 58.9% | 15788.6 | 42.8% | 10777.1 | 53.3% | 1192.9 | 36.0% | 0.0 | - | 31836.0 | 48.2% |
| Tanzania |  |  |  |  |  |  |  |  |  |  |  |  |
| 2010 | 211.3 | 1.1% | 1557.6 | 3.9% | 4201.6 | 0.9% | 237.2 | 1.2% | 0.0 | - | 6207.8 | 1.0% |
| 2011 | 2479.6 | 21.1% | 6969.4 | 40.4% | 16212.5 | 32.1% | 114.8 | 22.0% | 0.0 | - | 25776.2 | 32.1% |
| 2014 | 942.4 | 44.8% | 589.9 | 20.9% | 11030.4 | 40.0% | 238.6 | 46.0% | 0.0 | - | 12801.4 | 39.2% |
| Uganda |  |  |  |  |  |  |  |  |  |  |  |  |
| 2010 | 5886.3 | 4.2% | 948.6 | 4.4% | 7859.3 | 5.3% | 63.4 | 0.0% | 16.9 | - | 14774.6 | 4.8% |
| 2011 | 17718.1 | 33.3% | 7026.0 | 43.0% | 12483.8 | 42.7% | 52.1 | 49.8% | - | - | 37280.0 | 38.5% |
| 2013 | 8579.3 | 44.4% | 7054.8 | 44.5% | 12023.3 | 43.3% | - | - | - | - | 27657.4 | 43.8% |
| 2015 | 16760.3 | 41.5% | 10032.6 | 38.0% | 22465.9 | 52.9% | - | - | - | - | 49258.9 | 47.5% |
| Madagascar |  |  |  |  |  |  |  |  |  |  |  |  |
| 2010 | 1091.5 | 12.1% | 3659.5 | 8.1% | 1110.5 | 8.8% | 2870.0 | 3.4% | - | - | 8731.5 | 7.0% |
| 2011 | 228.8 | 53.9% | 2286.0 | 65.9% | 1733.7 | 23.0% | 1918.0 | 0.2% | - | - | 6166.5 | 19.5% |
| 2013 | 738.3 | 53.1% | 2501.6 | 36.6% | 987.0 | 35.9% | 490.4 | 25.1% | 170.3 | 4.1% | 4887.5 | 29.9% |
| 2015 | 821.9 | 4.8% | 3030.4 | 25.9% | 1188.7 | 4.9% | 364.9 | 0.7% | - | - | 5405.9 | 6.5% |
| nb. The number of AETD cannot be used as an indication of market size across countries, as the number depends on each survey’s sample size. It does not therefore show total market size for each country. | | | | | | | | | | | |  |
