# Supplementary material for: What happened to anti-malarial markets after the Affordable Medicines Facility-malaria pilot? Trends in ACT availability, price and market share from five African countries under continuation of the private sector co-payment mechanism
Source: Malar J. 2017 Apr 25;16:173. doi: 10.1186/s12936-017-1814-z (PMC5405529; doi:10.1186/s12936-017-1814-z)
Supplement: Supplementary file 2 — Additional file 2. Median private sector price for one AETD of QAACT and SP in 2009 USD, by country and year. [file 12936_2017_1814_MOESM2_ESM.docx]

**Additional file 2: Median private sector price for one AETD of QAACT and SP in 2009 USD, by country and year**

| **Country** | **Year** | **QAACT** | | | **SP** | | |
| --- | --- | --- | --- | --- | --- | --- | --- |
|  |  | **Number of products** | **Median price (2009 USD), [IQR]** |  | **Number of products** | **Median**  **price (2009 USD), [IQR]** |  |
| **Nigeria** | 2009 | 1001 | 4.04 [3.57] |  | 4061 | 0.54 [0.41] |  |
|  | 2011 | 1894 | 1.34 [1.61] | *** | 3045 | 0.43 [0.32] | *** |
|  | 2013 | 2530 | 1.30 [0.86] | ns | 2307 | 0.43 [0.31] | *** |
|  | 2015 | 8765 | 1.24 [0.86] | *** | 5745 | 0.37 [0.03] | *** |
| **Kenya** | 2010 | 742 | 2.57 [4.88] |  | 1235 | 0.51 [0.58] |  |
|  | 2011 | 2006 | 0.51 [0.45] | *** | 977 | 0.45 [0.67] | * |
|  | 2014 | 2209 | 1.24 [0.93] | *** | 1253 | 0.46 [0.41] | ** |
| **Tanzania** | 2010 | 286 | 5.01 [5.73] |  | 1441 | 0.86 [0.35] |  |
|  | 2011 | 1850 | 0.95 [0.63] | *** | 2018 | 0.95 [0.50] | * |
|  | 2014 | 3714 | 0.95 [0.48] | *** | 4909 | 0.72 [0.35] | ** |
| **Uganda** | 2010 | 583 | 2.79 [2.32] |  | 1186 | 0.70 [0.24] |  |
|  | 2011 | 3291 | 1.96 [1.65] | * | 1517 | 0.59 [0.19] | ns |
|  | 2013 | 3479 | 1.96 [1.05] | ns | 1246 | 0.49 [0.16] | *** |
|  | 2015 | 5278 | 1.48 [0.74] | *** | 2314 | 0.44 [0.15] | *** |
| **Madagascar** | 2010 | 385 | 0.14 [1.47] |  | 483 | 0.38 [0.19] |  |
|  | 2011 | 1091 | 0.61 [1.03] | *** | 474 | 0.39 [0.13] | ns |
|  | 2013 | 1424 | 0.58 [0.89] | *** | 647 | 0.35 [0.12] | *** |
|  | 2015 | 928 | 0.95 [1.76] | *** | 347 | 0.34 [0.41] | *** |
| ‡ median price in US dollars  Significance levels: *p<0.05; **p<0.01; ***p<0.001; ns= non-significant  Significance tests denote difference from previous year | | | | | | | |
